# Effect of anabolic hormone exposure during the backgrounding-phase in calf-fed steers of different mature sizes^1^

**DOI:** 10.1093/tas/txaa076

**Published:** 2020-06-06

**Authors:** Wes W Gentry, Ethan J Blom, Robbi H Pritchard, Kristin E Hales

**Affiliations:** 1 Department of Animal Science, South Dakota State University, Brookings, SD; 2 Annawan Cattle, Aurora, SD; 3 Department of Animal and Food Sciences, Texas Tech University, Lubbock, TX

**Keywords:** estrogen, feedlot, growth, implants, mature size

## Abstract

The mature size (MS) of cows in the United States is diverse, which leads to diversity in the MS of feeder cattle and hot carcass weights (HCW) of fed cattle. Cattle feeders must manage this inherent variation. Given that implants alter MS, they may be an effective tool to manage variation in HCW across groups of cattle. Two experiments were conducted to evaluate the interaction of MS and implant status in calf-fed steers. Steer calves from the same two sources were used in both experiments. Because breeding seasons were <60 d, it was assumed that weaning weight (WW) to a large extent reflected differences in MS. Smaller MS (SMS) and larger MS (LMS) steers were identified from the tails of the WW distribution. Within each MS group, steers were implanted with 20 mg estradiol benzoate and 200 mg progesterone (SS) or 14 mg estradiol benzoate and 100 mg trenbolone acetate (CH) on d 1 (Exp. 1), and nonimplanted (NI) or implanted with SS on d 2 (Exp. 2). In both experiments, a common terminal implant was used and steers were fed for 161 (Exp. 1) and 168 d (Exp. 2). Data were analyzed as a randomized complete block design with a 2 × 2 factorial treatment structure, with main effects of MS and implant. No MS × implant interactions were observed in either experiment (*P* ≥ 0.08). In both experiments LMS steers had heavier body weights (BW), HCW, and BW adjusted to 28% empty body fat (AFBW), greater average daily gain (ADG), dry matter intake (DMI), and lesser gain:feed (G:F) than SMS steers (*P* ≤ 0.02). No differences were evident in marbling score or Quality Grade distributions between SMS and LMS steers (*P* ≥ 0.13). In Exp. 1, no differences in growth performance, carcass traits, AFBW, or calculated frame size (FS) were observed for steers initially implanted with SS or CH (*P* ≥ 0.12). In Exp. 2, steers implanted initially with SS had heavier final BW, greater ADG and DMI (*P* ≤ 0.01), and similar G:F (*P* = 0.78) than NI steers. Steers initially implanted with SS had heavier HCW (*P* < 0.01), but no other differences in carcass characteristics were observed (*P* ≥ 0.23). Additionally, steers implanted with SS tended to have heavier AFBW (*P* = 0.07) and greater calculated FS (*P* = 0.05) than NI steers. Steers of different MS responded similarly to implants. Previous exposure to implants did not alter the response to the terminal implant. Estradiol increases the FS of steers; however, when similar doses of estradiol are compared, trenbolone acetate did not further increase FS (Exp. 1).

## INTRODUCTION

The mature size (MS) of the United States cowherd is diverse, which leads to diversity in feeder cattle MS and fed cattle hot carcass weights (HCW). MS can be ambiguous if not put into correct context. To clarify, MS is used herein to define body mass when steers fed similarly reach a market acceptable compositional endpoint. Frame size (FS) and MS are positively correlated (Tatum et al., 1986b), generally with one being indicative of the other. Cattle of varying frame score have inherent differences in performance potential and body composition at a given weight (Tatum et al., 1986a, 1986b, 1986c). Given that, in 1979 frame and muscle thickness scores were assigned as a method to value feeder cattle, and were later updated in 2000 (USDA, 2000; Grona et al., 2002).

At times, feedlots will sort feeder cattle to manage variation in MS (Armbruster et al., 2013). Sorting is accomplished based on weight and/or some assessment of MS with an assumption of age. However, MS can be altered by implants containing anabolic hormones (Preston, 1978; Loy et al., 1988). In fact, a meta-analysis concluded that aggressively implanted steers were 42 kg heavier at harvest than nonimplanted (NI) steers of similar body composition (Guiroy et al., 2002).

Implications of MS and implant status on growth of beef cattle have been documented; however, their potential interaction has only briefly been explored (Williams et al., 1987; Solís et al., 1989). Our hypothesis was that to reduce variation in HCW, perhaps a steer with a smaller MS (SMS) requires a more aggressive implant strategy than a contemporary with a larger mature size (LMS). The objectives of these experiments were to 1) examine the effects of backgrounding implant exposure and potency on animal growth performance, and carcass characteristics of steers of different MS and 2) determine if differences in HCW between smaller and LMS steers could be mitigated with backgrounding implant strategy.

## MATERIALS AND METHODS

Experiments were approved by the South Dakota State University Institutional Animal Care and Use Committee (Approval 17-004E and 17-077E, Exp. 1 and 2, respectively) and were conducted at the South Dakota State University Ruminant Nutrition Center. Experiment 1 was initiated on 22 December 2016 and terminated on 1 June 2017. Experiment 2 was initiated on 7 December 2017 and terminated on 24 May 2018. Angus and Angus × Simmental steers used in both experiments originated from the same two ranches in Western South Dakota. Calves were a product of <60 d breeding season and were castrated in early May at approximately 30–60 d of age during routine processes of branding and vaccinating on each ranch. Creep feed was not used on either ranch.

### Exp. 1

Spring born (March–April) steer calves were weaned and immediately shipped 588 km to the South Dakota State University Ruminant Nutrition Center. The following day, steers were individually weighed, vaccinated for clostridial (Ultrabac 7/Somubac, Zoetis Inc., Parsippany, NJ) and viral pathogens (Bovi-Shield Gold 5, Zoetis Inc.), treated with an anthelmintic for external parasites (Cydectin, Bayer, Shawnee Mission, KS), and were outfitted with a unique feedlot identification tag. The arrival body weight (BW) was considered as the weaning weight (WW).

Based upon prior experience with steer calves from the two sources, and because of a relatively short breeding season, it was presumed that cattle type within and between sources was similar. WW was used as a proxy for MS to segregate steers into SMS and LMS contemporary groups. Each MS group consisted of 64 steers from an overall population of 212 steers (WW = 281 kg, SD = 35.9 kg). WWs were 245 ± 1.9 kg and 321 ± 2.4 kg for SMS and LMS steers, respectively. Steers were allowed a 51-d acclimation period prior to initiation of the experiment. The initial 42 d of the acclimation period steers were enrolled in receiving experiments. Steers were sorted into MS groups and fed approximately two-times estimated maintenance energy intake (NASEM, 2016) of a common diet (13.4% CP, 1.79 Mcal/kg NEm) during the last 9 d of acclimation period to equalize gastrointestinal fill. No implants were used in the feedlot prior to trial initiation.

Steers were individually weighed to obtain a current BW to facilitate allotment to implant treatment (IMP). Steers were stratified by BW within MS group and assigned to IMP. Steers were then stratified by MS, IMP, and BW and assigned to replicate (*n* = 4). Steers were placed in pens (8 steers/pen) based upon MS × IMP × replicate, for a total of 16 pens (2 × 2 × 4). This procedure allowed for similar standard deviation of BW within pens.

On d 1, steers were weighed and implanted with either 20 mg estradiol benzoate + 200 mg progesterone (SS; Synovex S, Zoetis Inc.) or 14 mg estradiol benzoate + 100 mg trenbolone acetate (CH; Synovex Choice, Zoetis Inc.). These implants were chosen as they are similar in EB content; therefore, the implant comparison essentially evaluates whether or not the backgrounding implant formulation should contain trenbolone acetate (TBA). The BW recorded served as the initial BW for the experiment. The initial 84 d of the experiment represents distinct dietary and IMP management and will be referred to as the backgrounding-phase. This phase occurred while steers were grown from approximately 55% to 75% of final BW.

Outdoor pens were concrete surfaced with straw bedding and measured 7.6 × 7.6 m, with a 7.6 m fence-line feed bunk. Water tanks were located between adjacent pens and steers had ad libitum access to fresh water at all times. Diets fed during this experiment are reported in Table 1. The diet changed on d 36 because of evolving supplement inventory. Changes on d 70 and 91 were to increase dietary energy concentration. Feed deliveries were managed according to a clean bunk management system to approximate ad libitum intake and organized so that feed delivery sequence did not confound treatment. Steers were fed twice daily (starting at 0800, 1500 hours) in equal amounts to the nearest 0.45 kg (as-is basis) at each delivery. Feed ingredients were conveyed to the nearest 0.454 kg into a 2.4 m^3^ mixer (Roto-Mix, Dodge City, KS) and mixed for 4 min.

**Table 1.** Actual formulation and nutrient composition of diets fed in Exp. 1^*a,b*^

	Days of experiment			
Item	1–35	36–69	70–90	91–161
*n*, samples	5	5	3	10
Dry-rolled corn, %			49.67	67.98
High-moisture ear corn, %	51.52	54.70	24.55	
Dried distillers grains, %	16.50	14.59	11.91	
Modified distillers grains, %				19.56
Corn silage, %	25.00	25.68	8.90	
Sorghum silage, %				7.58
Liquid supplement 1^*c*^, %	5.00			
Liquid supplement 2^*d*^, %		5.03	4.97	4.88
Pelleted supplement^*e*^, %	1.98			
Dry matter, %	60.66	60.22	73.57	68.85
Crude protein, %	13.65	12.56	12.22	14.19
Neutral detergent fiber, %	21.31	21.06	15.00	14.37
Acid detergent fiber, %	9.62	9.92	6.06	5.40
Ash, %	3.73	3.66	2.87	3.51
Ether extract, %	4.11	4.07	3.86	4.35
NE_m_^*f*^, Mcal/kg	1.89	1.88	2.04	2.09
NE_g_^*f*^, Mcal/kg	1.24	1.23	1.37	1.40

^*a*^Dry matter basis.

^*b*^Calculated from weekly ingredient assays and batching records.

^*c*^Containted 39% crude protein as nonprotein nitrogen on a dry matter basis and provided vitamins and minerals to meet nutrient requirements (NASEM, 2016).

^*d*^Contained 39% crude protein as nonprotein nitrogen and 648 mg/kg monensin (Rumensin 90; Elanco Animal Health, Greenfield, IN) on a dry matter basis and provided vitamins and minerals to meet nutrient requirements (NASEM, 2016).

^*e*^Contained 1665 mg/kg monensin (Rumensin 90; Elanco Animal Health).

^*f*^NE_m_, net energy for maintenance; NE_g_, net energy for gain; calculated from tabular net energy values (Preston, 2016).

Individual feed ingredients were sampled weekly throughout the experiment. Feed samples were dried in a forced air oven at 60 °C until a constant weight was maintained to determine dry matter (DM) content, then ground (Wiley mill, model 4, Thomas Scientific, Swedesboro, NJ) to pass through a 1-mm screen. Ground samples were analyzed for DM (AOAC, 1990), crude protein (Kjedahl method; AOAC, 1990), neutral and acid detergent fiber (Goering and Van Soest, 1970), and ash (AOAC, 1990). Distillers grains samples (dried and modified) were additionally analyzed for ether extract content using an Ankom Fat Extractor (XT10; Ankom Technology, Macedon, NY). Tabular feed ingredient energy values (Preston, 2016) were used to estimate dietary net energy concentration. Dry matter intake (DMI), ingredient, and nutrient composition were calculated and summarized weekly using feed ingredient analyses and corresponding daily feed batching and delivery records.

Steers were weighed individually in the morning prior to feed delivery approximately every 28 d. Reported BW are shrunk (3%). Steers were implanted with 24 mg estradiol + 120 mg trenbolone acetate (Revalor-S, Merck Animal Health, Madison, NJ) on d 85. Steers were weighed in the morning and shipped on 1 June 2017 after 161 d on feed when mean back fat depth (BF) for the overall population was estimated to be 1.3 cm.

Steers were harvested at a commercial abattoir on 2 June 2017. Individual identity was tracked throughout the harvest and grading process. HCW was measured on the day of harvest. Dressing percent was calculated using shrunk (3%) final BW. Carcass data necessary to determine USDA yield and quality grade were obtained from the video image analysis system in the packing plant the following day. *Longissimus* muscle area (LMA), BF, marbling score, and percent kidney pelvic heart fat (KPH) from each side of the carcass were averaged for each carcass. Yield grade was calculated by using the USDA regression equation (USDA, 2016). Empty body fat (EBF) and weight at 28% EBF (AFBW) were estimated using equations from Guiroy et al. (2001) and compared with AFBW and FS reported by Fox et al. (1992). Additionally, estimated gain energy density (GED) was calculated from retained energy divided by observed average daily gain (ADG). Retained energy was estimated by using the feed for maintenance and feed for gain approach outlined by NASEM (2016) coupled with the weighted average feed NE values (Table 1) for the specific period in question. Net energy for maintenance was presumed to be equal to 0.077 Mcal per unit metabolic body size (MBS; Kleiber, 1961).

A necropsy was conducted on the two steers that died during the experiment, and in each case, it was determined that death was unrelated to treatment. One steer belonging to the SMS-CH treatment died on d 15 from hardware disease, and corresponding data were removed from the experiment. One steer belonging to the LMS–SS treatment was found dead in the home pen on d 115, from bloat. Differences in implant administration occurred prior to d 85, therefore, the data corresponding to the steer were included until d 84 and excluded post d 84.

Based upon AFBW distribution within MS groups, two LMS-NI, and one LMS-SS steer were identified as outliers of the MS classification. Steers were removed from carcass data but remained in performance data because individual contribution to pen DMI was not known.

Animal growth performance and carcass data were analyzed using the GLM procedure of SAS (SAS Inst. Inc., Cary, NC) as a completely randomized block design with a factorial arrangement of treatments. Main effects were 1) MS and 2) IMP. Replicate was considered a fixed blocking factor, which accounted for feed delivery sequence and feedlot location. The interaction of MS × IMP was included in the model. With a significant interaction, simple effect means were separated using a Fisher’s test. Pen served as the experimental unit for all analyses, excluding quality grade distribution. Quality grade distributions were analyzed as binomial proportions using the GLIMMIX procedure of SAS with the ILINK option where carcass was the experimental unit. For all analyses, effects were considered significant at *P* ≤ 0.05, and statistical trends at *P* ≤ 0.10.

### Exp. 2

Methods were similar to those described in Exp. 1 in terms of acclimation to facilities, feeding to equalize gastrointestinal tract fill, and segregation into SMS and LMS contemporary groups. Each MS group consisted of 80 steers from an overall population of 371 steers (WW = 280 kg, SD = 27.3 kg). Weaning weights were 253 ± 0.8 kg and 313 ± 1.6 kg for SMS and LMS steers, respectively.

Allotment procedures to IMP, replicate, and pens were similar to those described in Exp. 1 except that 5 replicates were used for a total of 20 pens (2 × 2 × 5). On d 1 steers were weighed to record an initial BW. On d 2 steers were either NI or implanted with SS. Once again, the backgrounding-phase occurred while the steers were grown from approximately 55% to 75% of final BW.

Diets fed during this experiment are reported in Table 2. On d 1 steers were fed the common diet used to equalize fill. The diet was changed on d 2, 71, and 78 to increase dietary energy density. On d 111 high-moisture corn and oat hay were used to replace high-moisture ear corn. Weighing schedule, laboratory assays, feed delivery, and bunk management were similar to those described in Exp. 1.

**Table 2.** Actual formulation and nutrient composition of diets fed in Exp. 2^*a,b*^

	Days of experiment			
Item	2–70	71–77	78–110	111–168
*n*, samples	10	1	5	9
Dry-rolled corn, %		13.70	37.54	34.44
High-moisture corn, %				34.34
High-moisture ear corn, %	62.58	60.30	39.10	
Dried distillers grains, %	16.51	17.25	18.27	18.08
Grass hay, %	15.76	3.69		
Oat hay, %				8.12
Liquid supplement^*c*^, %	5.15	5.06	5.09	5.02
Dry matter, %	69.98	70.48	74.30	79.52
Crude protein, %	12.91	13.22	13.82	13.94
Neutral detergent fiber, %	25.39	18.35	15.21	17.03
Acid detergent fiber, %	12.17	7.63	5.68	6.82
Ash, %	6.59	5.33	4.73	5.07
Ether extract, %	3.95	3.97	3.98	3.46
NE_m_^*d*^, Mcal/kg	1.85	1.97	2.05	2.10
NE_g_^*d*^, Mcal/kg	1.19	1.31	1.38	1.42

^*a*^Dry matter basis.

^*b*^Calculated from weekly ingredient assays and batching records.

^*c*^Contained 39% crude protein as nonprotein nitrogen and 648 mg/kg monensin (Rumensin 90; Elanco Animal Health, Greenfield, IN) on a dry matter basis and provided vitamins and minerals to meet nutrient requirements (NASEM, 2016).

^*d*^NE_m_, net energy for maintenance; NE_g_, net energy for gain; calculated from tabular net energy values (Preston, 2016).

On d 85, steers were implanted with 24 mg estradiol + 120 mg trenbolone acetate (Revalor-S; Merck Animal Health, Madison, NJ). Implant sites were appraised via ear palpation 28 d after each implant was administered by an individual who did not administer the previous implant. Abscess rate was 1.3% for steers receiving the SS implant and 1.9% for the terminal implant. Data were not excluded on the basis of implant abnormality. Steers were shipped on 24 May 2018 after 168 d on feed when mean BF depth for the overall population was estimated to be 1.3 cm. Steers were shipped to the same commercial abattoir, and procedures were similar to those described in Exp. 1. Carcass data were collected for all steers enrolled in the experiment.

Two steers died during the experiment, necropsies were performed, and it was determined death was unrelated to treatment. One steer belonging to the LMS-SS treatment was found dead in the pen on d 10, from intestinal hemorrhagic enteritis and interstitial pneumonia. Data unique to that steer were removed. One steer belonging to the SMS-SS treatment was found dead in the pen on d 128, from atypical interstitial pneumonia. Once again, differences in implant administration occurred prior to d 85, therefore, the data corresponding to the steer were included until d 84 and excluded from performance post d 84. Based on AFBW distribution within MS group, 1 steer from SMS-NI, SMS-SS, and LMS-SS, and three steers from LMS-NI were identified as outliers of the MS classification and data were handled as in Exp. 1.

Animal performance and carcass data were analyzed using the GLM procedure of SAS as a completely randomized block design with a factorial arrangement of treatments. Main effects were 1) MS and 2) IMP. Replicate was considered a fixed blocking factor, which accounted for feed delivery sequence. The interaction of MS × IMP was included in the model. With a significant interaction, simple effect means were separated using a Fisher’s test. Pen served as the experimental unit for all analyses, excluding quality grade distribution. Quality grade distributions were analyzed as binomial proportions using the GLIMMIX procedure of SAS with the ILINK option where carcass was the experimental unit. For all analyses, effects were considered significant at *P* ≤ 0.05, and statistical trends at *P* ≤ 0.10.

## RESULTS

The birthdates of steer calves (*n* = 89) from one of the sources used in Exp. 2 were obtained after the completion of the experiment. Days of age at weaning were 204.05 and 204.88 for SMS (*n* = 51) and LMS (*n* = 38) steers, respectively, and were not different (*P* = 0.88; SEM = 3.924).

Significant (*P* ≤ 0.05) interactions were not observed; therefore, main effect means of MS and IMP are reported. The backgrounding-phase represents when the main effect of IMP was applied and corresponds to d 1 to 84 in each experiment. The finishing-phase corresponds to d 85–161 (Exp. 1) or 168 (Exp. 2). These phases represent distinct differences in diet energy density and anabolic hormone exposure. All steers in both experiments received the terminal implant on d 85.

### Exp. 1

By design, initial BW was greater (*P* < 0.01) for LMS than SMS steers (Table 3). This difference persisted throughout the experiment, as observed in interim (d 84) and final BW (*P* < 0.01). During the backgrounding-phase, LMS steers had 8% greater (*P* = 0.03) ADG, 14% greater (*P* < 0.01) DMI, and tended (*P* = 0.06) to have 6% lesser G:F than SMS steers. Grams of DMI per kg of MBS and GED were not different for SMS and LMS steers during the backgrounding-phase (*P* ≥ 0.21). No differences (*P* ≥ 0.22) were observed for BW, ADG, DMI, G:F, or GED between steers implanted with SS or CH during the backgrounding-phase.

**Table 3.** Main effects of MS and backgrounding estradiol benzoate and progesterone or estradiol benzoate and trenbolone acetate on interim performance corresponding to management in steer calves fed for 161 d in Exp. 1^*a*^

	Mature size^*b*^			Implant^*c*^			Pooled
Item	SMS	LMS	*P* value	SS	CH	*P* value	SEM
Backgrounding-phase^*d*^							
*n*, steers	63	64	–	64	63	–	–
*n*, pens	8	8	–	8	8	–	–
Initial body weight, kg	287	360	<0.01	324	323	0.75	1.1
Interim body weight, kg	421	504	<0.01	463	461	0.55	2.8
Average daily gain, kg	1.59	1.71	0.03	1.66	1.64	0.64	0.033
Dry matter intake, kg	8.11	9.23	<0.01	8.77	8.57	0.22	0.104
Dry matter intake/MBS^*e*^, g/kg	99.4	97.4	0.22	99.4	97.4	0.22	1.04
Gain:feed	0.196	0.185	0.06	0.190	0.191	0.80	0.0037
GED^*f*^, Mcal/kg	3.82	3.99	0.21	3.95	3.87	0.55	0.090
Finishing-phase^*d*^							
*n*, steers	63	63	–	63	63	–	–
*n*, pens	8	8	–	8	8	–	–
Interim body weight, kg	421	503	<0.01	463	461	0.63	2.8
Final body weight, kg	562	651	<0.01	608	606	0.65	3.7
Average daily gain, kg	1.84	1.92	0.09	1.88	1.88	0.88	0.031
Dry matter intake, kg	11.12	12.42	<0.01	11.83	11.71	0.64	0.165
Dry matter intake/MBS^*e*^, g/kg	106.5	105.5	0.54	106.3	105.6	0.64	1.12
Gain:feed	0.166	0.155	<0.01	0.160	0.161	0.63	0.0018
GED^*f*^, Mcal/kg	5.47	5.82	0.01	5.66	5.62	0.69	0.073

^*a*^Shrink (3%) was applied to all body weights.

^*b*^SMS, smaller mature size; LMS, larger mature size.

^*c*^SS, implanted with 20 mg estradiol benzoate and 200 mg progesterone on d 1 and with 24 mg estradiol and 120 mg trenbolone acetate on d 85; CH, implanted with 14 mg estradiol benzoate and 100 mg trenbolone acetate on d 1 and with 24 mg estradiol and 120 mg trenbolone acetate on d 85.

^*d*^Backgrounding-phase, d 1–84; finishing-phase, d 85–161.

^*e*^MBS, metabolic body size, calculated as weight^3/4^ (Kleiber, 1961).

^*f*^GED, gain energy density, calculated as estimated retained energy divided by ADG.

During the finishing-phase, LMS steers exhibited a 12% greater (*P* < 0.01) DMI, tended to exhibit a 4% greater (*P* = 0.09) ADG, and had a 7% lesser (*P* < 0.01) G:F than SMS steers. However, DMI within context of MBS was not different between SMS and LMS steers (*P* = 0.54). Despite no differences in GED during the backgrounding-phase, GED was 6% greater (*P* = 0.01) for LMS steers than SMS steers during the finishing-phase. There were no differences observed between SS and CH treatment for any of the finishing-phase production variables (*P* ≥ 0.63).

Cumulative differences observed in the backgrounding and finishing-phases on both the live BW and carcass-weight basis are presented in Table 4. Compared with SMS steers, LMS steers had 6% greater (*P* ≤ 0.02) ADG, 13% greater DMI, 6% greater GED, and 7% lesser (*P* < 0.01) G:F on the live BW basis. DMI per MBS was not different between SMS and LMS steers (*P* = 0.34). On a carcass-adjusted basis, LMS steers exhibited a heavier final BW (*P* < 0.01), a 7% greater ADG (*P* = 0.01), and a 5% lesser G:F (*P* = 0.03) than SMS steers. No differences were evident in either the backgrounding or finishing-phases for the main effect of IMP, and no differences (*P* ≥ 0.12) were observed on the cumulative live BW or carcass-adjusted basis.

**Table 4.** Main effects of MS and backgrounding estradiol benzoate and progesterone or estradiol benzoate and trenbolone acetate on cumulative and carcass adjusted performance in steer calves fed for 161 d in Exp. 1^*a*^

	Mature size^*b*^			Implant^*c*^			Pooled
Item	SMS	LMS	*P* value	SS	CH	*P* value	SEM
*n*, steers	63	63	–	63	63	–	–
*n*, pens	8	8	–	8	8	–	–
Live weight basis							
Initial body weight, kg	287	360	<0.01	324	323	0.83	1.0
Final body weight, kg	562	651	<0.01	608	606	0.65	3.7
Average daily gain, kg	1.71	1.81	0.02	1.77	1.75	0.71	0.024
Dry matter intake, kg	9.55	10.76	<0.01	10.23	10.07	0.34	0.111
Dry matter intake/MBS^*d*^, g/kg	102.0	100.9	0.34	102.1	100.8	0.26	0.81
Gain:feed	0.179	0.168	<0.01	0.173	0.174	0.51	0.0015
GED^*e*^, Mcal/kg	4.61	4.87	<0.01	4.78	4.70	0.30	0.048
Carcass-adjusted basis							
Final body weight^*f*^, kg	553	645	<0.01	603	595	0.12	3.2
Average daily gain, kg	1.65	1.77	0.01	1.74	1.69	0.21	0.024
Gain:feed	0.173	0.165	0.03	0.170	0.168	0.58	0.0023

^*a*^Shrink (3%) was applied to all body weights.

^*b*^SMS, smaller mature size; LMS, larger mature size.

^*c*^SS, implanted with 20 mg estradiol benzoate and 200 mg progesterone on d 1 and with 24 mg estradiol and 120 mg trenbolone acetate on d 85; CH, implanted with 14 mg estradiol benzoate and 100 mg trenbolone acetate on d 1 and with 24 mg estradiol and 120 mg trenbolone acetate on d 85.

^*d*^MBS, metabolic body size, calculated as weight^3/4^ (Kleiber, 1961).

^*e*^GED, gain energy density, calculated as estimated retained energy divided by ADG.

^*f*^Calculated as HCW divided by 0.625.

LMS steers had greater HCW, REA, and yield grade than SMS steers (*P* ≤ 0.02). The SMS steers had a greater (*P* < 0.01) ratio of LMA to HCW. No differences were observed for dressing percent, marbling score, KPH, or individual quality grade categories (*P* ≥ 0.13) for SMS and LMS steers.

Estimated EBF, AFBW, equivalent shrunk body weight (EQSBW), and calculated FS were greater (*P* ≤ 0.01) for LMS compared with SMS steers (Table 5). SMS and LMS steers differed by approximately two frame scores. The percentage of carcasses grading High Choice tended (*P* = 0.08) to be greater for steers implanted with CH compared with SS. No other differences in carcass traits attributable to IMP were noted (*P* ≥ 0.12). Steers implanted with SS or CH had EBF, AFBW, and calculated FS that were not different (*P* ≥ 0.18).

**Table 5.** Main effects of MS and backgrounding estradiol benzoate and progesterone or estradiol benzoate and trenbolone acetate on carcass characteristics, EBF, and calculated frame score of steer calves fed for 161 d in Exp. 1

	Mature size^*a*^			Implant^*b*^			Pooled
Item	SMS	LMS	*P* value	SS	CH	*P* value	SEM
*n*, steers	63	60	–	61	62	–	–
*n*, pens	8	8	–	8	8	–	–
HCW, kg	346	403	<0.01	377	372	0.12	2.0
Dress^*c*^, %	61.47	61.95	0.14	61.86	61.56	0.32	0.205
Back fat thickness, cm	1.20	1.31	0.10	1.27	1.24	0.67	0.041
LMA, cm^2^	79.84	85.64	0.01	83.33	82.16	0.48	1.129
LMA/HCW, 6.45 cm^2^/45.4 kg	1.63	1.50	<0.01	1.56	1.56	0.96	0.018
Calculated yield grade	3.05	3.32	0.02	3.18	3.19	0.96	0.067
Marbling score^*d*^	563	569	0.69	556	576	0.18	9.8
Kidney pelvic heart fat	2.14	2.01	0.18	2.03	2.11	0.41	0.064
Quality grade							
Prime, %	0.00	0.00	–	0.00	0.00	–	–
High choice, %	1.68	3.17	0.59	0.00	4.87	0.08	1.990
Average choice, %	22.04	26.76	0.55	26.17	22.62	0.65	5.616
Low choice, %	65.17	51.42	0.13	55.40	61.18	0.52	6.384
Select, %	11.11	18.65	0.24	18.43	11.33	0.27	4.589
EBF^*e*^, %	29.34	30.61	0.01	29.97	29.98	0.98	0.259
AFBW^*e*,*f*^, kg	526	590	<0.01	562	554	0.19	3.6
EQSBW^*g*^, kg	270	301	<0.01	-	-	-	2.0
Calculated FS^*h*^	4.75	6.74	<0.01	5.87	5.62	0.18	0.117

^*a*^SMS, smaller mature size; LMS, larger mature size.

^*b*^SS, implanted with 20 mg estradiol benzoate and 200 mg progesterone on d 1 and with 24 mg estradiol and 120 mg trenbolone acetate on d 85; CH, implanted with 14 mg estradiol benzoate and 100 mg trenbolone acetate on d 1 and with 24 mg estradiol and 120 mg trenbolone acetate on d 85.

^*c*^Calculated as HCW divided by final body weight.

^*d*^Small^0^, 500, Modest^0^, 600.

^*e*^Estimated using equations of Guiroy et al. (2001).

^*f*^AFBW, final shrunk weight at 28% empty body fat.

^*g*^EQSBW, equivalent shrunk body weight (Tylutki et al., 1994).

^*h*^Estimated using final shrunk weight at 28% EBF and data from Fox et al. (1992).

### Exp. 2

Initial BW was greater (*P* < 0.01) for LMS than SMS steers by design (Table 6). ADG was not different (*P* = 0.14) between SMS and LMS steers during the backgrounding-phase. LMS steers had heavier (*P* < 0.01) interim BW (d 84) than SMS steers. DMI was 6% greater, G:F was 9% lesser, and GED was 11% greater (*P* < 0.01) for LMS than SMS steers during the backgrounding-phase. Similar to Exp. 1, DMI per kg of MBS was not different (*P* = 0.49) between SMS and LMS steers.

**Table 6.** Main effects of MS and backgrounding estradiol benzoate and progesterone on interim performance corresponding to management in steer calves fed for 168 d in Exp. 2^*a*^

	Mature size^*b*^			Implant^*c*^			Pooled
Item	SMS	LMS	*P* value	NI	SS	*P* value	SEM
Backgrounding-phase^*d*^							
*n*, steers	80	79	–	80	79	–	–
*n*, pens	10	10	–	10	10	–	–
Initial body weight, kg	308	371	<0.01	339	340	0.04	0.3
Interim body weight, kg	436	503	<0.01	466	474	0.01	1.7
Average daily gain, kg	1.52	1.57	0.14	1.50	1.59	0.01	0.021
Dry matter intake, kg	8.68	9.84	<0.01	9.14	9.38	0.01	0.055
Dry matter intake/MBS^*e*^, g/kg	102.4	103.0	0.49	101.8	103.7	0.03	0.53
Gain:feed	0.175	0.160	<0.01	0.165	0.170	0.08	0.0018
GED^*f*^, Mcal/kg	4.14	4.58	<0.01	4.40	4.32	0.31	0.050
Finishing-phase^*d*^							
*n*, steers	79	79	–	80	78	–	–
*n*, pens	10	10	–	10	10	–	–
Interim body weight, kg	436	503	<0.01	466	474	0.01	1.7
Final body weight, kg	587	660	<0.01	618	629	<0.01	1.9
Average daily gain, kg	1.79	1.87	0.01	1.81	1.84	0.24	0.018
Dry matter intake, kg	11.41	12.68	<0.01	11.82	12.27	<0.01	0.044
Dry matter intake/MBS^*e*^, g/kg	106.0	107.0	0.04	105.2	107.8	<0.01	0.31
Gain:feed	0.157	0.147	<0.01	0.154	0.150	0.15	0.0015
GED^*f*^, Mcal/kg	5.89	6.29	<0.01	5.99	6.19	0.05	0.064

^*a*^Shrink (3%) was applied to all body weights.

^*b*^SMS, smaller mature size; LMS, larger mature size.

^*c*^NI, nonimplanted during backgrounding-phase, implanted with 24 mg estradiol and 120 mg trenbolone acetate on d 85; SS, implanted with 20 mg estradiol benzoate and 200 mg progesterone on d 2 and with 24 mg estradiol and 120 mg trenbolone acetate on d 85.

^*d*^Backgrounding-phase, d 1–84; Finishing-phase, d 85–168.

^*e*^MBS, metabolic body size, calculated as weight^3/4^ (Kleiber, 1961).

^*f*^GED, gain energy density, calculated as estimated retained energy divided by ADG.

Initial BW was different (*P* = 0.04) between NI and steers implanted with SS; however, the difference is a function of a small variance due to the allotment procedure and is only 1 kg in magnitude (Table 6). Therefore, this difference is not biologically meaningful. During the backgrounding-phase steers implanted with SS had 6% greater ADG, 3% greater DMI, and 2% greater DMI per MBS compared with NI steers (*P* ≤ 0.03). Also, G:F tended to be 3% greater (*P* = 0.08) for steers implanted with SS compared with NI steers. Interim BW was greater (*P* = 0.01) for steers implanted with SS, while GED was not altered (*P* = 0.31).

During the finishing-phase, ADG was 4% greater (*P* < 0.01) for LMS than SMS steers. Final BW was also greater (*P* < 0.01) for LMS compared with SMS steers. LMS steers exhibited 11% greater (*P* < 0.01) DMI, and a 7% lesser (*P* < 0.01) G:F than SMS steers. DMI relative to MBS was greater (*P* = 0.04) for LMS compared with SMS steers; however, this difference was only 1 g/kg of MBS. GED was 7% greater (*P* < 0.01) for LMS compared with SMS steers during the finishing-phase.

Steers implanted with SS had ADG that did not differ (*P* = 0.24) from NI steers during the finishing-phase, and differences in interim BW persisted in final BW (*P* < 0.01). During the finishing-phase DMI and DMI per MBS of steers implanted with SS was 4% and 2% greater (*P* < 0.01), respectively, than NI steers. No difference (*P* = 0.15) was detected in G:F during the finishing-phase attributable to IMP. During the finishing-phase, GED was 3% greater (*P* = 0.05) for steers previously implanted with SS compared with NI.

Cumulative ADG and GED were greater (*P* < 0.01; Table 7) on a live BW and carcass-adjusted basis for LMS compared with SMS steers. LMS steers had greater (*P* < 0.01) DMI and DMI per kg MBS tended to be greater (*P* = 0.10) than SMS steers. SMS steers had greater (*P* < 0.01) cumulative and carcass-adjusted G:F than LMS steers. Carcass adjusted final BW was greater (*P* < 0.01) for LMS than SMS steers.

**Table 7.** Main effects of MS and backgrounding estradiol benzoate and progesterone on cumulative and carcass adjusted performance in steer calves fed for 168 d in Exp. 2^*a*^

	Mature size^*b*^			Implant^*c*^			Pooled
Item	SMS	LMS	*P* value	NI	SS	*P* value	SEM
*n*, steers	79	79	–	80	78	–	–
*n*, pens	10	10	–	10	10	–	–
Live weight basis							
Initial body weight, kg	308	371	<0.01	339	340	0.04	0.3
Final body weight, kg	587	660	<0.01	618	629	<0.01	1.9
Average daily gain, kg	1.66	1.72	<0.01	1.66	1.72	<0.01	0.012
Dry matter intake, kg	10.04	11.26	<0.01	10.48	10.83	<0.01	0.042
Dry matter intake/MBS^*d*^, g/kg	103.2	104.1	0.10	102.4	104.9	<0.01	0.34
Gain:feed	0.165	0.153	<0.01	0.159	0.159	0.78	0.0009
GED^*e*^, Mcal/kg	5.04	5.47	<0.01	5.23	5.28	0.24	0.033
Carcass-adjusted basis							
Final body weight^*f*^, kg	581	663	<0.01	616	628	<0.01	1.6
Average daily gain, kg	1.63	1.74	<0.01	1.65	1.71	<0.01	0.010
Gain:feed	0.162	0.154	<0.01	0.158	0.158	0.71	0.0009

^*a*^Shrink (3%) was applied to all body weights.

^*b*^SMS, smaller mature size; LMS, larger mature size.

^*c*^NI, nonimplanted during backgrounding-phase, implanted with 24 mg estradiol and 120 mg trenbolone acetate on d 85; SS, implanted with 20 mg estradiol benzoate and 200 mg progesterone on d 2 and with 24 mg estradiol and 120 mg trenbolone acetate on d 85.

^*d*^MBS, metabolic body size, calculated as kg of body weight^3/4^ (Kleiber, 1961).

^*e*^GED, gain energy density, calculated as estimated retained energy divided by ADG.

^*f*^Calculated as HCW divided by 0.625.

On a cumulative basis implanting steers with SS as an initial implant increased (*P* < 0.01) carcass-adjusted final BW, DMI, DMI per kg MBS, and ADG on a live and carcass-adjusted basis (Table 7). No differences between NI and SS were evident for GED, or G:F on a live or carcass-adjusted basis (*P* ≥ 0.24).

HCW, dressing percent, BF, LMA, and yield grade were greater (*P* < 0.01) for LMS compared with SMS steers. SMS steers had greater (*P* < 0.01) ratio of LMA to HCW and KPH compared with LMS steers. Marbling score between the two MS groups were not different (*P* = 0.77). No differences (*P* ≥ 0.27) were observed for the distribution of prime, high choice, average choice, low choice, or select quality grade between SMS and LMS steers.

Implanting steers with SS during the backgrounding-phase increased HCW compared with NI (*P* < 0.01). NI steers tended (*P* = 0.07) to have a larger LMA to HCW ratio compared with steers implanted with SS. No other differences (*P* ≥ 0.22) were observed attributable to IMP for any carcass characteristics other than HCW. EBF, AFBW, EQSBW, and calculated FS were greater (*P* < 0.01) for LMS steers than SMS steers. No difference in estimated EBF (*P* = 0.69) was observed between NI and SS. However, AFBW tended (*P* = 0.07) to be greater, and calculated FS was greater (*P* = 0.05) for steers implanted with SS compared with NI steers.

## DISCUSSION

From the available data, there was no age discrepancy in Exp. 2. Given that steers were allotted to MS group using the same procedure in Exp. 1, it is presumed MS was not cofounded by age. The absence of a significant MS × IMP interaction in the present experiments concur with results from previous experiments. Williams et al. (1987) fed small and large FS steers, and within FS compared NI with implantation with zeranol, which is a synthetic macrolide with estrogenic activity. Steers receiving zeranol implants were implanted on d 0 and 97, and all steers were fed for 175 d. Small and large FS steers responded similarly to zeranol in terms of growth performance and carcass characteristics. Solís et al. (1989) fed large and very-large FS steers for average of 182 d, and within FS steers were either NI or implanted twice with 36 mg zeranol, 72 mg zeranol, SS, or 36 mg zeranol + SS on d 0 and 90. The response by small and large FS steers was not different within each implant strategy. The present experiments, Williams et al. (1987), and Solís et al. (1989) all conclude that cattle of varying FS respond in a similar manner to growth promoting implants.

### MS—Exp. 1 and 2

During the backgrounding-phase, ADG was 8% greater for LMS steers in Exp. 1, while no difference was observed in Exp. 2 (Tables 3 and 6). Dietary energy differences between experiments during the backgrounding-phase were minimal (1.26 and 1.21 Mcal/kg NEg, in Exp. 1 and 2, respectively). The DMI effect of MS was similar across both experiments during the backgrounding-phase in that LMS steers consumed 13% to 14% more DM than SMS steers. DMI per MBS did not differ between SMS and LMS steers which infers that DMI was similar between the SMS and LMS steers in relation to their respective maintenance energy requirements.

The DMI response attributable to MS was of greater magnitude than the ADG response in both experiments. The discrepancy during the backgrounding-phase can be partially explained by differing GED. In Exp. 1, GED did not differ between SMS and LMS steers. However, in Exp. 2 LMS steers had greater GED than SMS steers. Therefore, in LMS steers the additional retained energy over that of SMS steers was predominantly in the form of adipose, which is less efficient on a G:F-basis than lean accretion (Owens et al., 1995).

Excluding moderate energy diets (Byers, 1980) and isolated instances where ADG was not different through 180 d of age (Cianzio et al., 1982), it is generally accepted that cattle of greater MS will have greater ADG, DMI, and G:F (Tatum et al., 1986b). The expected response in ADG, carcass-adjusted ADG, and DMI in relation to MS were observed in the current experiments. The SMS steers did exhibit greater G:F, and carcass-adjusted G:F than LMS steers (Tables 4 and 7). Trenkle (2001) reported that small FS cattle tend to be more efficient than large FS cattle that were not different in BF or yield grade. Several factors can influence G:F such as: maintenance energy requirements, DMI in excess of maintenance energy requirements, and composition of BW gain. Data were not collected in the present experiment that could infer any differences in maintenance energy requirements between SMS and LMS steers; however, given that GED was greater for LMS steers and DMI per MBS was similar to SMS steers, it is logical that those steers would be less feed efficient in terms of G:F. Feed efficiency is certainly of interest for cattle feeders. Its relative importance within a feeding enterprise is dependent upon business model, feed prices and fed cattle prices. Total live weight gain over the feeding period is always of importance to cattle feeders, and it should be acknowledged the LMS steers have that advantage over SMS steers.

LMS steers had greater BF in both Exp. 1 and 2 (Tables 5 and 8). This is contrary to our hypothesis. Tatum et al. (1986b) reported that the subcutaneous adipose depot represented a greater percent of all adipose depots in small FS compared with medium or large FS steers. Cianzio et al. (1982) reported similar findings between small and large FS steers. However, one must consider the accretion rate of subcutaneous adipose relative to other adipose depots. Late in the feeding period adipose accretion is rapid (Cianzio et al., 1982; Bruns et al., 2004). Williams et al. (1987) reported no difference in grams of protein deposition, but greater adipose deposition in large FS compared with small FS steers fed for 175 d. Additionally, Cianzio et al. (1982) reported that the allometric growth coefficient for subcutaneous fat of large FS steers was different than one, while this was not the case for small FS steers. In other words, the difference in BF in the present experiments may have manifested late in the feeding period. Regardless, the LMS steers had greater BF and a lesser G:F than SMS steers, which is a consequence of greater GED. This observation concurs with Hermesmeyer et al. (2000), who reported that steers fed to a targeted 1.4 cm BF had a 2.9% lesser G:F than steers targeted to achieve 1.0 cm BF.

**Table 8.** Main effects of MS and backgrounding estradiol benzoate and progesterone on carcass characteristics, estimated EBF, and calculated frame score of steer calves fed for 168 d in Exp. 2

	Mature size^*a*^			Implant^*b*^			Pooled
Item	SMS	LMS	*P* value	NI	SS	*P* value	SEM
*n*, steers	77	75	–	76	76	–	–
*n*, pens	10	10	–	10	10	–	–
HCW, kg	363	414	<0.01	385	392	<0.01	1.0
Dress^*c*^, %	61.92	62.75	<0.01	62.20	62.47	0.23	0.151
Back fat thickness, cm	1.39	1.58	<0.01	1.48	1.50	0.71	0.033
LMA, cm^2^	83.76	89.31	<0.01	86.18	86.88	0.34	0.495
LMA/HCW, 6.45 cm^2^/45.4 kg	1.62	1.53	<0.01	1.59	1.56	0.07	0.008
Calculated yield grade	3.15	3.45	<0.01	3.29	3.31	0.73	0.050
Marbling score^*d*^	618	621	0.77	623	616	0.54	7.6
Kidney pelvic heart fat	1.95	1.87	<0.01	1.92	1.90	0.22	0.013
Quality grade							
Prime, %	3.88	5.22	0.69	5.21	3.89	0.70	2.438
High Choice, %	13.00	12.01	0.86	10.56	14.45	0.48	3.892
Average Choice, %	40.07	36.15	0.62	41.93	34.29	0.33	5.601
Low Choice, %	39.19	38.52	0.93	34.33	43.38	0.26	5.696
Select, %	3.86	8.10	0.27	7.97	3.99	0.30	2.732
EBF^*e*^, %	30.72	32.23	<0.01	31.42	31.53	0.69	0.202
AFBW^*e*,*f*^, kg	529	581	<0.01	550	560	0.07	3.4
EQSBW^*g*^, kg	288	316	<0.01	–	–	–	1.7
Calculated FS^*h*^	4.82	6.48	<0.01	5.52	5.78	0.05	0.085

^*a*^SMS, smaller mature size; LMS, larger mature size.

^*b*^NI, nonimplanted during backgrounding-phase, implanted with 24 mg estradiol and 120 mg trenbolone acetate on d 85; SS, implanted with 20 mg estradiol benzoate and 200 mg progesterone on d 2 and with 24 mg estradiol and 120 mg trenbolone acetate on d 85.

^*c*^Calculated as HCW divided by final body weight.

^*d*^Small^0^, 500, Modest^0^, 600.

^*e*^Estimated using equations of Guiroy et al. (2001).

^*f*^AFBW, final shrunk weight at 28% empty body fat.

^*g*^EQSBW, equivalent shrunk body weight (Tyultki et al., 1994).

^*h*^Estimated using final shrunk weight at 28% EBF and data from Fox et al. (1992).

As would be expected from greater HCW, LMS steers had approximately 7% greater LMA than SMS steers (Tables 5 and 8). However, SMS steers had a greater LMA in relation to their respective HCW. Reinhardt et al. (2009) reported that medium FS steers and heifers exhibited a greater LMA per unit HCW than did large FS steers and heifers. This may be a result of the nonlinear relationship of LMA to HCW (Lawrence et al., 2008). Yield grade was greater for LMS than SMS steers. The difference is likely because LMS steers had greater BF, and also may partially be a result of the nonlinear relationship of LMA to HCW (Lawrence et al., 2008). Williams et al. (1987) and Trenkle (2001) report no difference in Yield Grade between small and large FS steers, but HCW in those studies were 20–100 kg lighter than those in the present experiments. LMS steers had heavier HCW, but LMA was not proportionally large enough to offset the increase in HCW relative to SMS steers. LMA is the only variable in the USDA equation that is associated with a reduction in yield grade (USDA, 2016); therefore, the LMS steers may be at a disadvantage to SMS steers in terms of Yield Grade.

Marbling score was not different between the 2 MS groups for either experiment. This is in agreement with the published literature (Cianzio et al., 1982; Williams et al., 1987; Trenkle, 2001). Because marbling develops during early growth (Bruns et al., 2004), and DMI per MBS did not differ during the backgrounding-phase, we did not anticipate any differences in marbling score. Furthermore, backgrounding-phase ADG relative to each MS must have been sufficient to support intramuscular adipose accretion.

Estimated EBF was greater for LMS than SMS steers (Tables 5 and 8). In the equation used to estimate EBF, the largest coefficient is associated with BF (Guiroy et al., 2001). Therefore, differences in BF are likely being magnified in estimated EBF. Nonetheless, this was not anticipated, and it was expected that EBF would be similar on a common harvest day for steers that were different in MS. Solís et al. (1989) reported no difference in measured EBF between large and very-large FS steers at harvest. However, EBF estimated via D_2_O dilution was lesser for very-large FS steers at the initiation of the experiment. In that experiment actual EBF was 21.9% and 23.2% at harvest for large and very-large steers respectively. However, those steers were harvested at a lesser chemical maturity than the steers were harvested in the present experiments. Given the rate of adipose accretion discussed previously, perhaps if the steers used in Solís et al. (1989) were fed for a longer duration, EBF may have been greater for the very-large FS steers.

Adjusting final BW to a common EBF produced expected results; AFBW was greater for LMS than SMS steers in both experiments (Tables 5 and 8). This observation is a cornerstone in discussing cattle of various MS. Cattle of all MS are capable of reaching a similar body composition in a similar timeframe; however, LMS cattle have heavier BW than SMS at a similar compositional endpoint. Furthermore, LMS steers had a greater calculated FS than SMS steers. This observation validates that the experiment was initiated with steers of differing MS.

Initially, it was puzzling as to why the LMS steers exhibited a greater GED than SMS steers at a constant day on feed. If the equivalent weight system proposed by Tylutki et al. (1994) and used by NASEM (2016) is considered with the initial BW and AFBW, it is evident that the LMS steers began the experiment at a greater EQSBW, or in other words were nearer to chemical maturity (28% EBF). As an animal becomes more mature, adipose comprises a larger portion of daily gain and GED increases. Greater GED then leads to greater EBF at harvest, all of which were observed with LMS steers compared with SMS steers. These observations are in contrast with the FS experiments of the 1980s; however, in those experiments the authors (Tatum et al., 1986a) disclose that FS was cofounded with breed (*n* = 20) because cattle were purchased from various sources. Growth performance expectations formed from those experiments fit cattle sourced from sale barns with an unknown background quite well. Crude size may be indicative of growth potential when other relevant biological parameters are unknown; however, our understanding of true growth differences strictly attributable to MS may be lacking.

### Backgrounding Implant—Exp. 1

Estradiol benzoate is 71.4% estradiol on a molecular weight basis. As mentioned previously, the two initial implants used in Exp. 1 are similar in EB content. The implant comparison essentially evaluates whether or not the backgrounding implant formulation should contain TBA. No differences in backgrounding-phase growth performance or DMI were observed whether steers were implanted with SS or CH (Table 3).

All steers received a common terminal implant and responded similarly during the finishing-phase regardless of the initial implant (Table 3). There is a misconception that once cattle are implanted, they do not respond as well to subsequent implants. This is not the case when estrogenic implants are used (Mader et al., 1994; Pritchard et al., 2003), and in the current experiment the TBA in the CH implant did not decrease performance relative to SS implanted steers. There was no difference in cumulative live, or carcass-adjusted animal growth performance (Table 4). Herschler et al. (1995) compared steers implanted with SS, or CH to control steers fed for 140 d. Steers implanted with SS and CH had 15% and 18% greater ADG, and 6% and 8% greater G:F than control steers.

Nielson et al. (2016) implanted steers with CH followed by 28 mg EB + 200 mg TBA (Synovex Plus; Zoetis Inc.), or SS followed by 24 mg estradiol + 120 mg TBA (Revalor-S). No differences in growth performance were observed, which is in agreement with the present experiment. In large pens, Folmer et al. (2009) implanted steers with SS, or 16 mg estradiol + 80 mg TBA (Revalor-IS; Merck) as an initial implant, with both groups implanted with Revalor-S as a terminal implant. Steers implanted with Revalor-IS tended to have a greater carcass-adjusted final BW and HCW. Only cumulative performance is reported, so it is unclear whether the difference in BW started to accumulate early because of the initial implant, or if steers implanted with Revalor-IS responded more favorably to the terminal implant.

No differences were evident in carcass characteristics for steers implanted initially with SS or CH other than more SS steers tended to grade High Choice (Table 5). However, this is likely a type I error because of the limited number of observations (Galyean and Wester, 2010), the difference was due to three carcasses. In this experiment, one steer represents approximately 1.6% of the main effect population, which is almost as great as the SEM for that variable. Herschler et al. (1995) observed no differences in carcass characteristics between steers implanted with SS or CH for the duration of a 140-d feeding period, which is in agreement with the present experiment.

Steers implanted with SS or CH had similar estimated EBF (Table 5). Similarly, Folmer et al. (2009) observed no difference in estimated EBF between steers implanted initially with SS or Revalor-IS. Loy et al. (1988) found that NI steers, steers implanted once or twice with SS, or steers implanted once or twice with 36 mg zeranol had EBF that were not different after 189 d on feed. In the present experiment, implanting steers with SS or CH during the backgrounding-phase did not yield differences in EBF, AFBW or calculated FS. It seems that both implants were equally effective in promoting growth during the backgrounding-phase and did not alter the response to the terminal implant. More than one implant strategy likely generates desirable outcomes in respect to production and marketing goals (Pritchard, 1994; Parr et al., 2006; Prouty and Larson, 2010; Nielson et al., 2016).

### Backgrounding Implant—Exp. 2

During the background phase, ADG was increased 6% in steers implanted with SS relative to NI steers. An increase of greater magnitude was expected. According to data compiled by Duckett el al. (1997), the use of a single SS implant should yield a 15% ADG response compared with a NI counterpart. A query conducted on the Merck and Texas Tech University North American TBA Implant Database reported a 12% ADG response comparing steers implanted with SS compared with NI (Merck and TTU, 2019). In those experiments a single SS implant was administered for the duration of the trial; whereas, in the present experiment the SS was administered on d 2 and the terminal implant was administered on d 85. Pritchard (1994) compared various implant strategies in steers fed for 140 d, all implanted steers received an initial and d 70 implant. Steers initially implanted with SS had 12% greater ADG through the initial 70 d compared with NI steers. Loy et al. (1988) observed an 8% increase in ADG in steers implanted with SS over NI steers during the initial 84 d of a 189-d experiment. The latter two experiments discussed represent a similar implant evaluation timeframe as the current experiment, and those studies (Loy et al., 1988; Pritchard, 1994) noted a greater ADG response from the SS implant.

Implants increase DMI as well as ADG, so when a desired ADG response is not observed one has to evaluate whether a DMI response was evident. Implanting steers with a SS can increase DMI by 6% (Anderson and Botts, 1995). In the current experiment, steers implanted with SS had 3% greater DMI than NI steers. Duckett et al. (1997) reported a 4% increase, Pritchard (1994) reported a 5% increase, and Loy et al. (1988) reported no increase in DMI of steers implanted with SS relative to NI steers. In the present experiment, steers implanted with SS had 2% greater DMI when scaled MBS, which has also been demonstrated by Pritchard (1998) using other implants. Clearly, DMI is increased because of reasons other than increased BW, perhaps by the anabolic profile supplied by the implant. Given these data, it is likely that DMI was not limiting the ADG response in steers implanted with SS. Estrogenic implants slightly increase maintenance energy requirements (Rumsey et al., 1980; Rumsey and Hammond, 1990), but BW gain and nitrogen retention increases more rapidly in steers administered an estrogenic implant compared with NI steers, when DMI approached ad libitum (Rumsey and Hammond, 1990). It is unclear why only a 6% increase in ADG was observed in the current experiment.

Steers implanted with SS tended to have 3% greater G:F than NI steers. Implanting steers with SS have resulted in a 5% to 10% increase in G:F (Loy et al., 1988; Pritchard, 1994; Duckett et al., 1997). The G:F response in the present experiment was lesser in magnitude because of the lesser ADG response. In the present experiment, GED was not different during the backgrounding-phase. Pritchard (1998) demonstrated a decreased GED when estradiol/TBA combination implants were used. Implants effectively increase the FS of cattle (Preston, 1978; Loy et al., 1988), which means that implanted steers are in a leaner stage of growth relative to NI steers at a similar point in time.

No difference was observed in finishing-phase ADG after steers had received the terminal implant. As discussed previously there is a misconception that once implanted, steers do not respond as well to subsequent implants. DMI and DMI per MBS was greater for steers implanted with SS. The payout of a SS implant is approximately 120 d (Mader, 1997). Given that all steers were reimplanted 84 d after implantation of the SS, it is likely at the beginning of the finishing-phase steers implanted with SS were exposed to more anabolic than NI steers. This may be why the DMI response persisted into the finishing-phase. No difference in G:F were observed during the finishing-phase, again lending evidence that previous implantation does not hamper the effect of subsequent implants in a well-designed strategy (Table 6). During the finishing-phase, steers implanted with SS had a greater GED than NI steers. It seems that both implant groups had maximized their lean growth potential, and because steers implanted with SS had greater DMI the additional retained energy was deposited as adipose tissue. Daily fat deposition was increased in steers implanted twice with 36 mg zeranol compared with NI steers in a 175-d feeding period reported by Williams et al. (1987).

Steers implanted with SS maintained the interim BW advantage over NI steers, as illustrated by final BW and carcass-adjusted final BW being greater (Table 7). Byers (1980) observed that steers implanted with diethylstilbestrol during the growing and finishing-phase had more kg of protein in the empty body than steers NI during the growing-phase but implanted with diethylstilbestrol during finishing. In the current experiment, steers implanted with SS initially had greater cumulative ADG, DMI, DMI per MBS, and carcass adjusted ADG than NI steers. No differences were observed in carcass-adjusted or live BW basis G:F, or GED between NI and SS steers. The absence of a difference in GED and G:F between NI and SS steers is attributable to a similar EBF, and a similar spread in final BW vs AFBW. The additivity of sequential implantation has been demonstrated by Mader et al. (1994) and Pritchard et al. (2003). Duckett and Andrea (2001)reported that weight gain from implanting is additive throughout all phases of beef production, which is supported by the results of the present experiment.

Steers implanted with SS during the backgrounding-phase had heaver HCW than NI steers, despite receiving a common terminal implant (Table 8). Duckett and Andrae (2001) report a 4.8% increase in HCW over NI steers when one estrogen + androgen combination implant is used. Additionally, they report a 6.6% increase in HCW over NI steers when a reimplant strategy is used involving an initial estrogenic implant followed by an estrogen + androgen combination implant. The difference relative to NI controls between these two implant strategies is approximately 2%, which is similar to the response in the current experiment.

The tendency observed for NI steers to have a greater LMA/HCW ratio is biologically irrelevant given that the difference was only 0.2 cm^2^/kg of HCW. Delaying or lowering the potency of the initial implant has lessened the decrease in marbling score attributable to implanting (Pritchard, 1998; Bruns et al., 2005; Smith et al., 2018). Therefore, we were particularly interested in marbling score responses attributable to altering implant exposure during the backgrounding-phase. While all implant dosages have decreased marbling score to some extent, evidence exists that implies TBA may be more detrimental to marbling score or percentage of carcass grading choice (Reinhardt and Wagner, 2014). Although, according to the data of Herschler et al. (1995) an implant containing 60 mg of estradiol or a SS implant depressed marbling relative to NI steers, while steers implanted with 300 mg TBA had marbling scores not different than NI steers. However, no difference in marbling score in the present experiment was observed (Table 8). Perhaps DMI was sufficient to meet the additional growth demand resulting from the implant, without impeding development of intramuscular fat depots. Depression in marbling scores associated with implanting may be related to energy intake at the time of implanting (Bruns et al., 2005). Implants increase lean growth potential, and if DMI is not sufficient to meet that increased growth potential while maintaining intramuscular adipose accretion, marbling scores may be decreased.

No difference in estimated EBF was observed for NI steers or steers implanted with SS during the backgrounding-phase (Table 8). It has been well documented that steers are capable of achieving a similar degree of EBF despite various implant strategies (Loy et al., 1988; Hutcheson et al., 1997; Bruns et al., 2005). Implanting steers with SS initially tended to increase AFBW compared with NI steers and is in agreement with published literature (Loy et al., 1988; Hutcheson et al., 1997; Bruns et al., 2005; Smith et al., 2018). Whether the quantitative measure of MS is AFBW (Loy et al., 1988; Hutcheson et al., 1997; Bruns et al., 2005; Smith et al., 2018), or FS measured as hip height (Preston, 1978; Loy et al., 1988) it is well documented that implants increase both units of measure.

Improving carcass uniformity should allow feedlot operators to market cattle more precisely. With low variation in a pen of cattle, the mean HCW can approach the heavyweight discount without having a high percentage of carcass that receive the discount. Variation in MS exists in all groups of cattle, and we hypothesized that variation in HCW could be reduced by implanting SMS cattle twice, and LMS only once. The following data consider each carcass as an individual. As a baseline, NI steers that only received a terminal implant had HCW of 385 ± 3.8 kg, and steers implanted with SS during the backgrounding-phase and received a terminal implant had HCW of 392 ± 3.7 kg. Remember that the NI and SS groups were composed of SMS and LMS steers. A hypothetical population was formed with SM-SS and LG-NI steers. The hypothetical population had HCW of 388 ± 3.5 kg. Using the SEM as a metric, the variation was slightly reduced but is likely not of large enough magnitude to be biologically relevant. It should be acknowledged, however, that if a greater gain response was observed with the SS then the variation in the hypothetical population would have been reduced further.

## IMPLICATIONS

SMS and LMS steers did not differ in response to implants used in these experiments. The growth response expected from LMS cattle was observed. In general, LMS steers consumed more feed, and exhibited increased rates of gain. However, SMS steers were more feed efficient, with greater G:F. The latter was because LMS steers were more mature at the onset of the experiment despite being similar in age. Future experiments should investigate growth differences in cattle with various MS when genetics, age, and lifetime plane of nutrition are controlled for. It is imperative to note that the calves being similar in age was cornerstone to the sorting strategy employed in these experiments which assigned steers to MS groups based on weaning BW. In populations where age in not known, this sorting strategy will likely not yield desired results because BW is indicative of more than MS.

Steers of different MS responded similarly to implants, which allows designing implant strategies to be less complicated. Implanting steers with an estrogenic implant during the backgrounding-phase increased HCW without decreasing marbling scores, and previous exposure to anabolic hormones did not alter the response to the terminal implant. The latter lends evidence that implant responses are additive throughout phases of production in a well-designed strategy. Estradiol increases the FS of steers; however, when similar doses of estradiol are compared, trenbolone acetate does not further increase FS.
